# Case Report: SARS-CoV-2 as an unexpected causal agent of predominant febrile hepatitis

**DOI:** 10.12688/f1000research.52929.2

**Published:** 2021-07-07

**Authors:** Paraskevas Filippidis, Francois van Ouwenaller, Alberto Cerutti, Anaïs Geiger-Jacquod, Christine Sempoux, Giuseppe Pantaleo, Darius Moradpour, Frederic Lamoth

**Affiliations:** 1 Infectious Diseases Service, Lausanne University Hospital and University of Lausanne, Lausanne, Vaud, 1011, Switzerland; 2 Internal Medicine Service, Lausanne University Hospital and University of Lausanne, Lausanne, Vaud, 1011, Switzerland; 3 Service of Gastroenterology and Hepatology, Lausanne University Hospital and University of Lausanne, Lausanne, Vaud, 1011, Switzerland; 4 Service of Clinical Pathology, Institute of Pathology, Lausanne University Hospital and University of Lausanne, Lausanne, Vaud, 1011, Switzerland; 5 Service of Immunology and Allergy, Department of Medicine, Lausanne University Hospital and University of Lausanne, Lausanne, Vaud, 1011, Switzerland; 6 Swiss Vaccine Research Institute, Lausanne University Hospital and University of Lausanne, Lausanne, Vaud, 1011, Switzerland; 7 Institute of Microbiology, Lausanne University Hospital and University of Lausanne, Lausanne, Vaud, 1011, Switzerland

**Keywords:** COVID-19, hepatology, liver, serology

## Abstract

**Background: **Respiratory symptoms and pneumonia are the predominant features of Coronavirus disease 2019 (COVID-19) due to emerging SARS-CoV-2 virus, but extrapulmonary manifestations are also observed. For instance, some degree of liver injury has been described among patients requiring hospital admission for severe COVID-19. However, acute febrile hepatitis as an initial or predominant manifestation of COVID-19 has been rarely reported.

**Case presentation:** A 34-year-old man without underlying medical conditions presented with fever of unknown origin for two weeks in the absence of respiratory symptoms or other complaints. Laboratory testing revealed isolated acute hepatitis, for which an extensive microbiological work-up did not reveal identification of the causal agent. PCR testing for SARS-CoV-2 on a nasopharyngeal swab was negative on two occasions and initial serology for SARS-CoV-2 (at 15 days from symptoms onset) was also negative. However, repeated SARS-CoV-2 serological testing at 30 days demonstrated seroconversion leading to the diagnosis of COVID-19-related hepatitis. The patient's condition progressively improved, while transaminases steadily declined and eventually returned back to normal within 30 days.

**Conclusions:** We describe here a unique case of SARS-CoV-2 isolated febrile hepatitis in a young and previously healthy man, which was diagnosed by demonstration of seroconversion, while PCR screening was negative. This case report highlights the role of repeated serological testing for the diagnosis of extrapulmonary manifestations of COVID-19.

## Background

Severe acute respiratory syndrome Coronavirus 2 (SARS-CoV-2), the etiological agent of Coronavirus disease 2019 (COVID-19) can cause a wide spectrum of clinical presentations ranging from mild flu-like illness to severe pneumonia with acute respiratory distress syndrome ^1^. While the pandemic is ongoing, atypical presentations with extrapulmonary organ involvement, including heart, kidneys, skin, nervous system, hepatobiliary and gastrointestinal tract are increasingly recognized ^2^. Liver injury with mild or moderate elevation of transaminases has been observed in about half of patients requiring hospital admission for severe COVID-19 ^3, 4^. However, acute febrile hepatitis as a predominant manifestation of COVID-19 has been rarely reported and has been mainly observed among patients with underlying liver diseases and/or concomitant signs of upper or lower respiratory tract involvement with diagnosis established by polymerase chain reaction (PCR) in nasopharyngeal swabs ^5– 7^. We report here a case of a young previously healthy man presenting with febrile hepatitis as a unique clinical manifestation, for which diagnosis of COVID-19 was established by seroconversion.

## Case presentation

**Figure 1.**
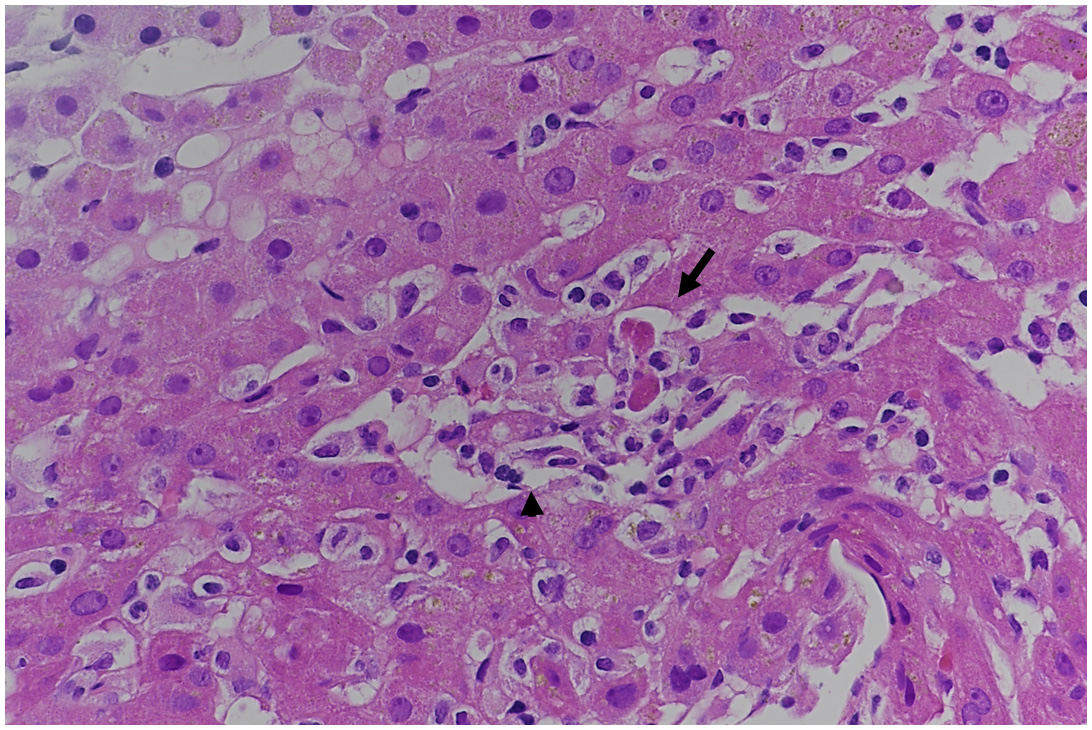
Histologic findings in liver biopsy. Mild lobular hepatitis with few apoptotic bodies (arrow) surrounded by mononuclear inflammatory cells (arrowhead). Hematoxylin-eosin staining, original magnification X400.

**Figure 2.**
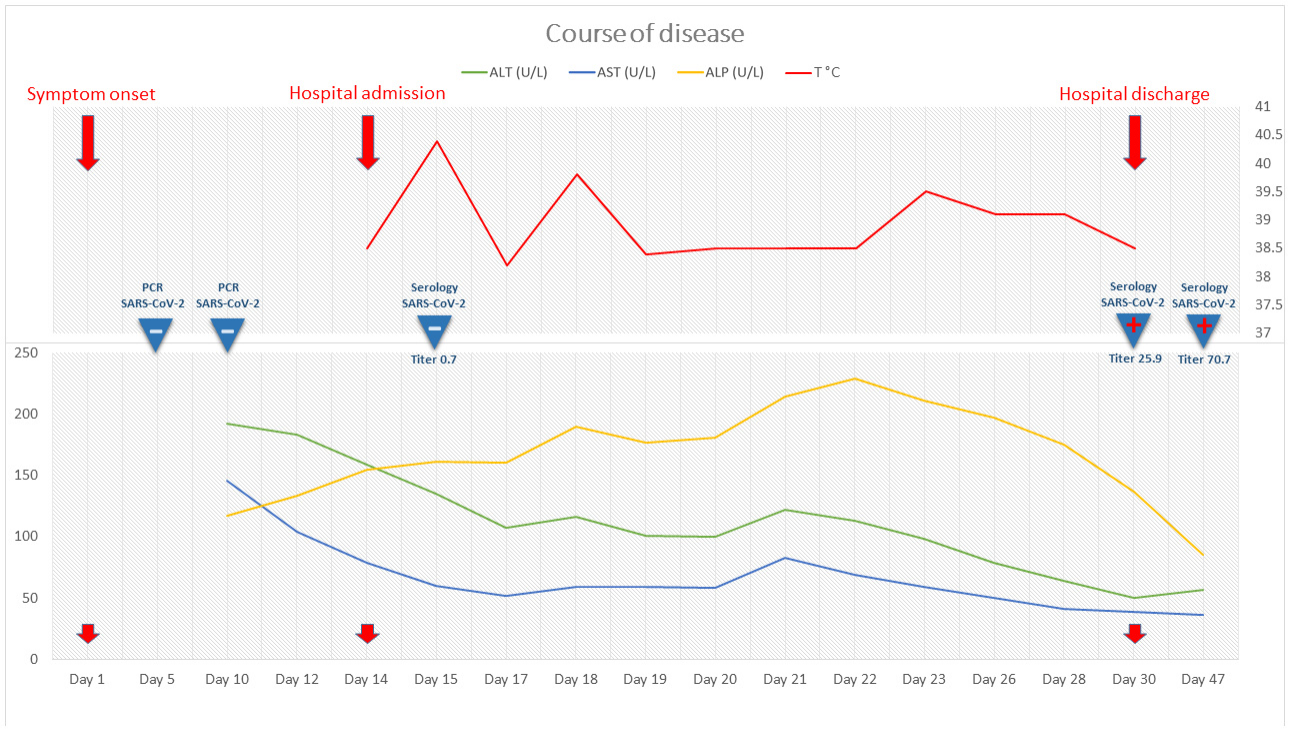
Clinical course of the disease for the present case report. Evolution of fever and liver function tests as well as results of SARS-CoV-2 PCR and serology. Serology testing for SARS-CoV-2 was performed by the Luminex S protein trimer IgG assay, as previously described ^8^. Results are expressed in mean fluorescence intensity ratio with a positive cut-off of 6. ALT, alanine aminotransferase; AST, aspartate aminotransferase; ALP, alkaline phosphatase; γ-GT, γ-glutamyltranspeptidase.

A 34-year-old previously healthy Caucasian man was admitted to our tertiary care hospital for investigation of fever of unknown origin. He had presented night fever up to 39.5°C with rare chills, night sweats and moderate headache for two weeks prior to admission. His symptoms responded to acetaminophen (500 mg once or twice per day on demand during 14 days; cumulative dose of 13.5 g upon admission) and nonsteroidal anti-inflammatory drugs (nimesulid 100 mg once per day on demand during 14 days; cumulative dose of 900 mg upon admission). During the first week after fever onset, he had also presented with a mild sore throat and dry cough, which had completely resolved at the time of admission. Exposure history was remarkable with travel to a Greek island one month ago and a superficial scratch from his neighbor’s cat two months ago. He declared no use of herbal medications or other dietary supplements before or after symptom onset. Upon admission, his vital signs and physical examination were unremarkable. Laboratory tests showed systemic inflammation with leukocytosis (10.5 G/l), thrombocytosis (501 G/l) and elevated inflammatory markers (C-reactive protein 126 mg/l, ferritin 695 µg/l), as well as acute hepatitis with a three-fold increase of transaminases, a two-fold increase of alkaline phosphatase and a four-fold increase of gamma-glutamyltranspeptidase. Total bilirubin was normal. Cervical, thoracic and abdominal computed tomography was unremarkable. PCR for SARS-CoV-2 on a nasopharyngeal swab was negative on two occasions. Serology for SARS-CoV-2 performed at admission (i.e. at 15 days from fever onset) was negative using the Luminex S protein trimer IgG assay, as previously described ^8^. Serologies for hepatitis A, B, C and E viruses, Epstein-Barr virus, cytomegalovirus and human immunodeficiency virus were negative. Further microbiological diagnostic work-up, including serological testing for bartonellosis, Q fever, brucellosis, tularemia, Lyme disease, rickettsial diseases, toxoplasmosis, syphilis and leptospirosis was negative. Autoimmune and metabolic causes of hepatitis were also excluded, including negative antinuclear antibodies and rheumatoid factor, normal complement values and total immunoglobulin G, negative complete autoantibody panels for autoimmune hepatitis and vasculitis, as well as normal copper and ceruloplasmin values and normal thyroid tests. We consequently performed a percutaneous liver biopsy, which showed nonspecific acute lobular hepatitis with no evidence of endotheliitis and negative immunohistochemical staining for herpes viruses ( Figure 1). Cultures of the liver tissue were sterile and broad-spectrum bacterial (16S rDNA), fungal (18S rDNA) and mycobacterial PCRs, as well as specific PCRs for *Brucella* spp., *Bartonella* spp., *Coxiella burnetii*, were all negative. Transthoracic echocardiography found no endocarditis-related abnormality. Positron emission tomography (PET) revealed moderate hypermetabolism of the posterior naso-oropharynx and numerous hypermetabolic cervical lymph nodes. However, naso-oropharyngeal endoscopy and cervico-facial magnetic resonance imaging found no structural abnormality. Serologies for hepatitis A, B, C and E viruses, cytomegalovirus and Epstein-Barr virus were tested again at a two-week interval and remained negative. However, repeated SARS-CoV-2 serology (i.e. at 30 days from the onset of fever) turned out clearly positive. This result was confirmed on a subsequent serum sample collected one week later and showing increasing titers. Retrospective SARS-CoV-2 PCR testing in serum and in liver tissue was negative. As illustrated in Figure 2, the patient’s condition progressively improved despite persistent low-grade fever at discharge, while transaminases steadily declined and eventually returned back to normal within 30 days. Fever had completely resolved after six weeks of follow-up.

## Discussion and conclusions

Investigations of acute hepatitis includes a diagnostic work-up for classical hepatotropic viruses and some other well known, albeit rarer, infectious agents causing liver injury. We report here a clinical observation raising attention to a novel pathogen that should be considered and actively searched for in such situations: the emerging SARS-CoV-2 virus.

While concomitant liver injury has been commonly observed among patients with severe COVID-19 pneumonia ^3, 4^, clinical presentation of COVID-19 with hepatitis as the predominant feature has been rarely reported ^5– 7^. All these cases had positive PCR for SARS-CoV-2 in nasopharyngeal swabs at admission, three had concomitant pulmonary involvement and two had predisposing liver conditions (e.g. chronic hepatitis C or previous liver transplantation).

Unlike previous reports ^5, 7^, our patient had no underlying liver disease and presented isolated fever and hepatitis with no radiological evidence of pulmonary involvement and negative SARS-CoV-2 PCR in nasopharyngeal swabs. Seroconversion occurring in a timeframe that was consistent with the course of the disease, after exclusion of other infectious and non-infectious causes of hepatitis, led to the diagnosis of COVID-19-related hepatitis in this case.

Interestingly, seroconversion occurred relatively late (between two and four weeks from fever onset). In a recent population-based seroprevalence study, the serological method used in this case (i.e. Luminex S protein trimer IgG assay) showed a sensitivity of 97% and a specificity equal or above 97%, when performed by day 15 from symptom onset ^8^. However, modest or delayed antibody responses (up to day 20) have been reported ^9^, which could be more frequently observed in young patients with less severe forms of the disease. While specific data about the antibody response in patients with extrapulmonary manifestations of COVID-19 are lacking, the present case report highlights the role of serology for the diagnosis of these atypical forms presenting later in the course of the disease, when respiratory symptoms are absent or not predominant, and the need for repeated serological testing (up to 3–4 weeks from symptoms onset) in case of high clinical suspicion and/or absence of alternative diagnosis.

In the present case, attempts to demonstrate the presence of the virus in blood or liver tissue by PCR were unsuccessful. Indeed, the rate of positive SARS-CoV-2 PCR in non-respiratory samples, such as blood or deep-organ tissues, is notoriously low ^10^. Although the nature of liver injury in COVID-19 remains to be elucidated, abnormal inflammatory response, rather than direct viral cytotoxicity, is suggested as the main pathophysiological mechanism of hepatitis ^11^. A case series of patients deceased from COVID-19 showed lobular hepatitis in 50% of liver autopsies, but found no correlation between histologic findings and a positive PCR assay for SARS-CoV-2 on liver tissue ^12^.

In conclusion, we report a case of SARS-CoV-2 infection with acute febrile hepatitis as a predominant manifestation. Importantly, this case highlights the need to include COVID-19 in the differential diagnosis of primary hepatitis while the pandemic is ongoing, and the crucial role of repeated serological testing (up to three to four weeks from symptoms onset) for the identification of such atypical extrapulmonary manifestations of the disease.

## List of abbreviations

SARS-CoV-2            Severe acute respiratory syndrome coronavirus 2

COVID-19                Coronavirus disease 2019

PCR                           Polymerase chain reaction

## Consent

Written informed consent for publication of their clinical details and/or clinical images was obtained from the patient.

## Data availability

All data underlying the results are available as part of the article and no additional source data are required.
