# Supplementary material for: Case Report: SARS-CoV-2 as an unexpected causal agent of predominant febrile hepatitis
Source: F1000Res. 2021 Jul 7;10:400. Originally published 2021 May 18. [Version 2] doi: 10.12688/f1000research.52929.2 (PMC8630549; doi:10.12688/f1000research.52929.2)
Supplement: Peer review history

# Peer review: Reviewer response for version 2

**DOI:** 10.5256/f1000research.58315.r99842

**Published:** 2021-11-29

**Authors:** Marco Merli

**Affiliations:** 1 Infectious Diseases, ASST Grande Ospedale Metropolitano Niguarda, Milan, Italy

The authors describe a case of SARS-CoV-2-related mild acute hepatitis occurring late after acute flu-like sydrome, which was identified only by late positive serology. The case is well described, as are the investigations performed. The missed virological diagnosis further supports the immunological mechanism of liver damage, since an efficient immune response could have led to early viral clearance. Actually mild hepatitis is very common in hospitalized patients with COVID-19-related respiratory failure, but little is avaible in non-hospitalized patients who are less frequently investigated with liver function tests.

Are enough details provided of any physical examination and diagnostic tests, treatment given and outcomes?

Yes

Is the case presented with sufficient detail to be useful for other practitioners?

Yes

Is sufficient discussion included of the importance of the findings and their relevance to future understanding of disease processes, diagnosis or treatment?

Yes

Is the background of the case’s history and progression described in sufficient detail?

Yes

Reviewer Expertise:

Infectious Diseases, with particular attention to immunocompromised hosts, intensive care unit and surgical patients

I confirm that I have read this submission and believe that I have an appropriate level of expertise to confirm that it is of an acceptable scientific standard.

# Peer review: Reviewer response for version 2

**DOI:** 10.5256/f1000research.58315.r89203

**Published:** 2021-07-08

**Authors:** Werner Albrich

**Affiliations:** 1 Division of Infectious Diseases and Hospital Epidemiology, Kantonsspital St. Gallen, St. Gallen, Switzerland

The authors have addressed my suggestions appropriately. Thank you.

Are enough details provided of any physical examination and diagnostic tests, treatment given and outcomes?

Partly

Is the case presented with sufficient detail to be useful for other practitioners?

Yes

Is sufficient discussion included of the importance of the findings and their relevance to future understanding of disease processes, diagnosis or treatment?

Yes

Is the background of the case’s history and progression described in sufficient detail?

Partly

Reviewer Expertise:

Infectious diseases

I confirm that I have read this submission and believe that I have an appropriate level of expertise to confirm that it is of an acceptable scientific standard.

# Peer review: Reviewer response for version 1

**DOI:** 10.5256/f1000research.56261.r85690

**Published:** 2021-06-28

**Authors:** Werner Albrich

**Affiliations:** 1 Division of Infectious Diseases and Hospital Epidemiology, Kantonsspital St. Gallen, St. Gallen, Switzerland

The authors describe the rare case of a 34 yo male with acute Covid-19 infection which manifested mainly as acute and self-limiting hepatitis aside from mild upper respiratory symptoms, treated with acetaminophen and NSAIDs. Despite nasopharyngeal testing with PCR and liver biopsy for SARS-CoV-2 PCR, only seroconversion for SARS-CoV-2 was positive. Extensive work-up of alternative causes of the acute hepatitis were all negative.

**Minor comments:**

- Should provide more detail on doses and frequency of acetaminophen and NSAIDs used for his hepatitis.
- Did he take any herbal medications for the cold symptoms?
- Provide more detail of tests for autoimmune and metabolic causes of the hepatitis.
- Was there any pathological evidence of endotheliitis on liver biopsy?
- Generally speaking, I agree with the authors’ conclusions but would rephrase the title and manuscript text in that the hepatitis was not isolated but predominant.

Are enough details provided of any physical examination and diagnostic tests, treatment given and outcomes?

Partly

Is the case presented with sufficient detail to be useful for other practitioners?

Yes

Is sufficient discussion included of the importance of the findings and their relevance to future understanding of disease processes, diagnosis or treatment?

Yes

Is the background of the case’s history and progression described in sufficient detail?

Partly

Reviewer Expertise:

Infectious diseases

I confirm that I have read this submission and believe that I have an appropriate level of expertise to confirm that it is of an acceptable scientific standard, however I have significant reservations, as outlined above.

# Response

**Published:** 2021-06-30

**Authors:** Paraskevas Filippidis

**Affiliations:** Centre Hospitalier Universitaire Vaudois, Switzerland

Dear reviewer, 

 Thank you for your precious feedback about our article and for the questions you submitted. Please find below point-to-point answers to your questions. 

- *Should provide more detail on doses and frequency of acetaminophen and NSAIDs used for his hepatitis.*
- **Response:** Acetaminophen was taken at a dose of 500 mg once or twice per day on demand during 14 days, namely a cumulative dose of 13.5 g upon admission, while the nonsteroidal anti-inflammatory drug nimesulid was taken at a dose of 100 mg once per day on demand during 14 days, namely a cumulative dose of 900 mg upon admission. In fact, these medications were rather used for the febrile syndrome than for his hepatitis and doses were relatively low to explain the liver damage observed. In addition, liver enzymes progressively normalized despite the continuous administration of the above mentioned medication to treat fever.
- *Did he take any herbal medications for the cold symptoms?*
- **Response:** No herbal medications or dietary supplements were taken by the patient. 
- *Provide more detail of tests for autoimmune and metabolic causes of the hepatitis.*
- **Response: **Autoimmune and metabolic diagnostic work-up included antinuclear antibodies, rheumatoid factor, complement, total immunoglobulin G, complete autoantibody panels for autoimmune hepatitis and vasculitis, copper and ceruloplasmin values and normal thyroid tests, which were all normal. Liver imaging was also normal.
- *Was there any pathological evidence of endotheliitis on liver biopsy?*
- **Response: **No pathological evidence of endotheliitis was found in liver biopsy.

- *Generally speaking, I agree with the authors' conclusions but would rephrase the title and manuscript text in that the hepatitis was not isolated but predominant.*
- **Response:** We rephrased the title’s manuscript as to indicate a predominant rather than isolated character of patient's febrile hepatitis.

On behalf of my co-authors, 

 Paraskevas Filippidis
